# Data on cell viability of human lung fibroblasts treated with polyphenols-rich extract from *Plinia trunciflora* (O. Berg) Kausel)

**DOI:** 10.1016/j.dib.2016.01.028

**Published:** 2016-01-22

**Authors:** Caroline Calloni, Luciana Fernandes Silva Santos, Luana Soares Martínez, Mirian Salvador

**Affiliations:** Laboratório de Estresse Oxidativo e Antioxidantes, Instituto de Biotecnologia, Universidade de Caxias do Sul (UCS), 95070-560 Caxias do Sul, RS, Brazil

**Keywords:** Mitochondria, Jaboticaba, *Plinia trunciflora*, Phenolic compounds, Amiodarone

## Abstract

Jaboticaba (*Plinia trunciflora* (O. Berg) Kausel) is a Brazilian native berry, which presents high levels of polyphenols. Here we provide data related to the effects of the polyphenols-rich extract from jaboticaba on the cell viability, mitochondrial complex I (nicotinamide adenine dinucleotide/CoQ oxidoreductase) activity and ATP biosynthesis of human lung fibroblast cells (MRC-5) treated with amiodarone. The data presented in this article demonstrate that the polyphenols-rich extract from jaboticaba was able to reduce cell death as well as the decrease in complex I activity and ATP biosynthesis caused by amiodarone in MRC-5 cells.

**Specifications Table**

**Table:** 

Subject area	Biochemistry
More specific subject area	Cell culture
Type of data	Graph
How data was acquired	Data were acquired by spectrophotometry and luminescence, using a microplate reader (Victor-X3, Perkin-Elmer, Finland).
Data format	Analyzed data.
Experimental factors	Human lung fibroblast cells (MRC-5) were grown in DMEM medium and pretreated with two non-cytotoxic concentrations (50 and 250 µg mL^−1^) of polyphenols-rich extract from jaboticaba (PEJ) for 1 h and after exposed to 100 µM of amiodarone for 24 h.
Experimental features	Cell viability was evaluated using MTT (3-(4,5-dimethylthiazol-2-yl)-2,5-diphenyltetrazolium bromide) assay, complex I activity was determined by using Complex I Enzyme Activity Microplate Assay Kit (Mitoscience, Abcam, Cambridge, MA, USA), and ATP levels were assayed with the Cell-Titer-Glo® kit assay (Promega,Madison, WI).
Data source location	Jaboticaba fruits were collected in Passo Fundo, Rio Grande do Sul (28° 18′ 45.75″ S; 52° 24′ 57.64″ W), Brazil. Experiments were performed in the laboratory of Oxidative Stress and Antioxidants at the University of Caxias do Sul, Rio Grande do Sul, Brazil.
Data accessibility	The data are provided in this article.

## Value of the data

• These data bring perspectives for further studies, which will contribute to futher understand about the mechanisms of AMD toxicity in human lung cells.
• The finding that PEJ was effective in reducing the damage caused by AMD may contribute to the development of therapies to reduce the pulmonary toxicity of AMD.
• These data open new perspectives to understand the biological effects of the phenolic compounds found in jaboticaba berries.

## 1 Data

**Fig. 1:**
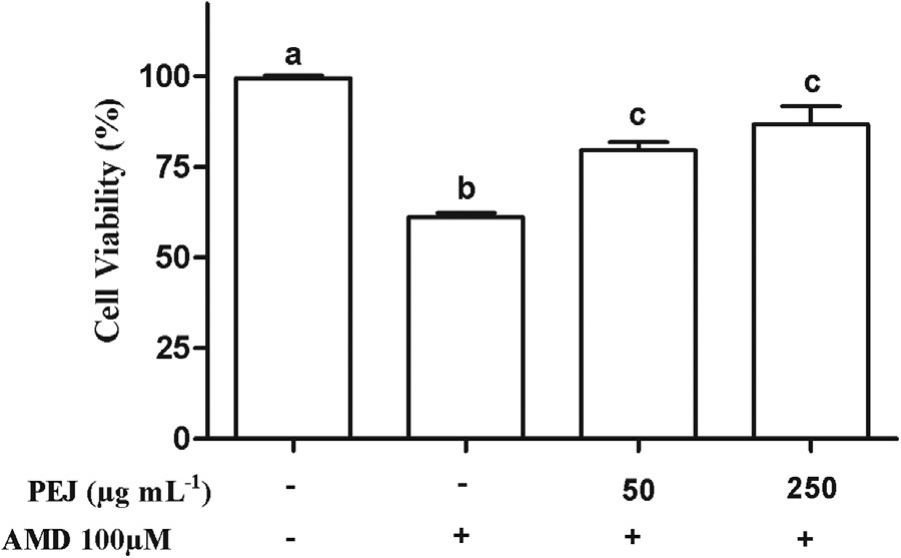
Viability of MRC-5 cells pretreated with PEJ (50 and 250 μg mL^−1^) and/or AMD (100 μM). The results are expressed as mean±SD from at least three independent experiments. Different letters indicate significantly different values among the treatments according to the analysis of variance (ANOVA) and Tukey׳s post-hoc test. Statistical significance was determined at *p*<0.05.

**Fig. 2:**
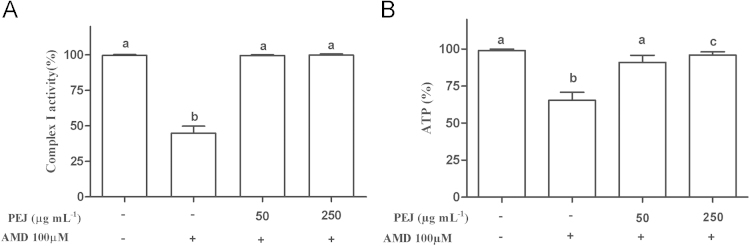
Complex I activity (A) and ATP levels (B) in MRC-5 cells pretreated with PEJ (50 and 250 μg mL^−1^) and/or AMD (100 μM). The results are expressed as mean±SD from at least three independent experiments. Different letters indicate significantly different values among the treatments according to the analysis of variance (ANOVA) and Tukey׳s post-hoc test. Statistical significance was determined at *p<*0.05.

Amiodarone (AMD) is widely used to treat cardiac arrhythmia, however it presents some adverse effects, which include pulmonary toxicity [1]. Jaboticaba present high levels of polyphenols, including cyaniding-3-*O*-glucoside and kaempferol [2], which are recognized as molecules capable of modulating pathways that defines mitochondrial processes of eukaryotic cells, such as complex I activity [3]. In these data article, we evaluate the effect of polyphenols-rich extract from jaboticaba (PEJ) in human lung fibroblast cells in presence and absence of AMD. These data show that AMD induced a reduction of 40% in MRC-5 cell viability, a decrease of 55% in mitochondria complex I activity and committed 35% of the ATP biosynthesis. Polyphenols-rich extract from jaboticaba was able to prevent the decrease in cell viability (Fig. 1) as well as to reduce the decrease in complex I activity and ATP biosynthesis (Fig. 2A and B) induced by AMD.

## 2 Experimental design, materials and methods

### 2.1 Plant material and extract preparation

Jaboticaba fruits were collected in Passo Fundo, Rio Grande do Sul, Brazil (28° 18′ 45.75″ S; 52° 24′ 57.64″ W). Polyphenol-rich extract from jaboticaba (PEJ), rich in cyanidin-3-*O*-glucoside and kaempferol, was prepared as already described by us [2].

### 2.2 Cell culture

Human lung fibroblasts cells (MRC-5 **–** ATCC® CCL-171™) were cultured in Dulbecco’s Modified Eagle Medium (DMEM), supplemented with heat inactivated 10% fetal bovine serum, penicillin (100 UI mL^−1^) and streptomycin (100 μg mL^−1^). Cells were maintained at 37 °C under an atmosphere of 5% CO_2_ with 90% relative humidity.

### 2.3 Mitochondrial dysfunction assay

To evaluate complex I activity of the mitochondrial electron transport chain, 1×10^7^ cells were treated for 1 h with PEJ (50 and 250 μg mL^−1^). After the removal of PEJ, the cells were exposure to 100 μM of AMD for 24 h. The cells were washed with cold phosphate-buffered-saline (PBS), and then scraped and homogenized in ice-cold PBS buffer. The cells were assayed for complex I activity using the Complex I Enzyme Activity Microplate Assay Kit (Mitoscience, Abcam, Cambridge, MA, USA) according to the manufacturer׳s instructions. The results were presented as a percentage of the control.

To verify a possible alteration in the ATP production, 5×10^4^ cells mL^−1^ were treated with 50 and 250 μg mL^−1^ of PEJ for 1 h. After removal of the PEJ, cells were exposure to 100 μM of AMD for 24 h. After removal of treatment, cells were assayed for their ATP concentrations using the Cell-Titer-Glo® assay (Promega, Madison, WI) according to the manufacturer׳s instructions. The results were presented as a percentage of the control.

### 2.4 Cell viability assay

Cell viability was evaluated using MTT (3-(4,5-dimethylthiazol-2-yl)-2,5-diphenyltetrazolium bromide) assay [4]. For this propose, cells were seeded at a density of 1×10^5^ cells mL^−1^ and grown for 24 h in DMEM medium. After that, cells were treated with PEJ (50 and 250 µg mL^−1^) for 1 h, and then they were exposed to 100 µM for 24 h. Thereafter, MTT solution (1 mg mL^−1^) was added, and cells were cultured for 3 h. The supernatant was discarded, and formazan precipitates were dissolved in 150 μL of DMSO per well. The optical density of the resultant solution was measured with a microplate reader (Victor-X3, Perkin Elmer, Finland) at 517 nm. The viability of the cells was expressed as a percentage of the control.

### 2.5 Statistical analysis

Statistical analysis was performed using the software SPSS 21.0 (SPSS Inc., Chicago, IL, USA). Data are expressed as the mean±standard deviation (SD) from at least three independent experiments and were determined to be parametrical by using the Kolmogorov–Smirnoff test. Data were subjected to the analysis of variance (ANOVA) and Tukey׳s post hoc test. Statistical significance was determined at *p*<0.05.

## Conflicts of interest

None.
